# Competitive Debate as Innovation in Gamification and Training for Adult Learners: A Conceptual Analysis

**DOI:** 10.3389/fpsyg.2021.666871

**Published:** 2021-12-15

**Authors:** Guillermo A. Sánchez Prieto, María José Martín Rodrigo, Antonio Rua Vieites

**Affiliations:** ^1^Management Department, Business and Economics School, Universidad Pontificia Comillas, Madrid, Spain; ^2^Quantitative Methods Department, Business and Economics School, Universidad Pontificia Comillas, Madrid, Spain

**Keywords:** competitive debate, lifelong learning, training, communication skills, teaching innovation, development, adult learners, gamification

## Abstract

Adult learners demand teaching innovations that are ever more rapid and attractive. As a response to these demands and the challenges of skills training, this article presents a conceptual analysis that introduces competitive debate as an impact training model. The aim is to learn whether debate can be considered to fall within the frame of gamification, so that the full potential of debate as gamification can be exploited. There is a significant research gap regarding competitive debate as a game, with the training mechanics for adult learners remaining practically unexplored. Through a conceptual analysis of game, game experience, and gamification, and their respective characteristics, we conclude that competitive debate is an ideal instrument for gamification.

## Introduction

As a phenomenon, rapid current change is, and should be, managed by leaders with a great personal capacity for communication (Gillen and Carroll, 1985; Mumford et al., 2007). Within the framework of leadership training as delivered by business schools, there exists a special sensibility toward communication skills training (Pfeffer and Fong, 2002), and the paradigm change to a less directive and more collaborative leadership now requires, more than ever, the reinforcement of social skills for communicating to and influencing others (Sobral and Furtado, 2019). Along with this new leadership style, according to Roberts et al. (2012), comes a new generation's particular aptitude for learning and being trained through play. Without active learning opportunities, students do not internalize skills, which makes it more difficult, if the methodology for developing leadership competences is lacking, to train them in skills (Sobral and Furtado, 2019). In this dual context of a demand for learning skills and a change in leadership paradigms, gamification is the ideal response to a learning environment that seeks to be active, visual, and playful, in line with the profile of millennials (Marcinkus Murphy, 2012; Roberts et al., 2012). In brief, there exists a need for active resources for training adults and future leaders in communication, with the meta-analysis literature recommending research into gamification use with adults and people over 30 so as to learn whether it can work (Klock et al., 2020). A shift in gamification activities from virtual to physical is required, as well as the promotion of social interaction and collaboration in gamification (Koivisto and Hamari, 2019; Rapp et al., 2019); following this line, several researchers also propose low technology learning environments or even the complete absence of digital resources (Zainuddin et al., 2020). Competitive debate may be a good way of fulfilling all these requirements, but we should first confirm whether, from a conceptual point of view, it can strictly be considered gamification. This paper offers a first proposal, arising from the taxonomy used by certain theorists' systematic mapping on gamification (de Sousa Borges et al., 2014); our proposed solution is to introduce debate mechanics as a further gamification tool for adults.

Gamification through e-learning has been shown to be effective for teaching and learning purposes at any educational stage, for subjects as diverse as foreign languages, entrepreneurship, and communication skills in general (Antonaci et al., 2015). Gamification has recently been used to train future entrepreneurs, making it possible to affirm that gamification strategies do indeed make an effective contribution to the reinforcement of the internalization learning processes (Antonaci et al., 2015).

The most common channel for applying gamification is the web environment, whether virtual or mobile, with most current research focusing on such digital spaces, and rarely on a non-virtual format with a physical presence (Antonaci et al., 2015)—although Kapp (2012a) and Girardelli et al. (2016), do claim the compatibility of the gamification concept with educational face to face presence, particularly for skills training and more specifically for communication skills. Furthermore, according to Osipov et al. (2015), for training in *soft skills*, digital gamification brings certain limitations with regard to the real time that participants may be connected with a game—which, according to their experience, is ~20 mins. Thus, they conclude that, for this type of training, in-person gamification is more effective and meaningful for learning than that developed *via* the e-learning format.

The object of this paper is not to explore the virtues or risks of game play as a learning methodology. Our aim is to verify whether or not the mechanics of competitive debate may be considered gamification for training purposes, whilst also asking whether the mechanics of competitive debate may be used by organizations to train adult professionals, to drive certain attitudes, and to acquire knowledge. We will emphasize the benefits that have been demonstrated with the use of these techniques in training processes, such as time-saving, an increase in commitment, and an impact on income that amounts to $30 m in corporations such as L'Oreal, IBM, Cisco, Deloitte, and other Fortune 500 companies (Larson, 2019).

Bibliographical research reveals that there are no published works relating to debate usage as a gamified training technique, whether for communication skills training or for the professional environment in general; neither has the question of whether competitive debate might fit into the gamification framework been addressed. The only available research that discusses debate as training mechanics for professionals is Delgado and Repiso (2013), who employed competitive debate as a strategy with State Department professionals in the United States administration. But, in Benton's work, the question of whether debate may be considered gamification remains unanswered.

The fact that some authors consider gamification as a strong potential training technique for professionals suggests that if debate were to be classified as gamification, it would open up a wide range of possibilities for research into training models. Bibliographical research does suggest that several surrounding conceptual areas have indeed been widely discussed, including:

- debate and its pedagogical consequences—its positive and negative effects, teaching techniques for debate, applications, etc., particularly in the educational arena (Colbert, 1987; Allen et al., 1997; Pernecky, 1997; Bellon, 2000; Perandones et al., 2018)
- competitive debate: evidence, its relationship with public speaking, training, coaching debate, strategy and competitive debate philosophy; in brief, competition-related aspects, as found in debating handbooks (Cirlin, 1999; Cattani, 2003; Huber and Snider, 2006; Sánchez Prieto, 2017)
- training methods in communication skills in the field of professional or educational organizations, and their techniques. Several papers, for example, establish the relationship between preparation and performance in public speaking (Menzel and Carrell, 1994), while others, such as Ebrahimi et al. (2019) explain in their metanalysis, seek to reduce the fear of public speaking. The authors of the present paper have published a debating model, a solution proposal paper for teaching and grading mechanics in the subject of Human Resources, within the degree of Business Administration (Sánchez Prieto, 2017). This paper explains that such a debate would comprise a competition between adults who were discussing an issue that would be of interest to a company. Through exposition, rebuttal, and counter rebuttals shifts, a jury would evaluate the speakers' proposed solutions as well as their communication skills and decide the winning team, with the debate teams receiving points from a jury and competing against other teams through different rounds.

There are, in summary, many publications on debate techniques, debate pedagogy, and professional training. But the research gap lies in the opportunity presented by the combination of these three elements as a gamification technique applied to continuous training, since conclusive research on the issue does not exist.

The innovative technique of competitive debate, applied to training in both professional interpersonal communication skills and other soft skills, therefore merits proposal. The idea would be, in short, to use the academic competitive debate as a training method in a gamified training context. This first article is the first of a research series, part of a more ambitious broader project that would seek to test the limits of debate as a gamification methodology; our purpose is to determine whether or not debate can be an effective model for training professionals in communication skills.

In short, this investigation seeks to discern whether competitive academic debate may be conceptually considered to be a gamification training mechanism. It is not our aim to determine whether gamification is positive or negative, to identify its effects, or to define gamification. Neither is it our intention to unpick conceptual controversies about what may or may not be gamification, or to review the literature.

In order to achieve its goal, this paper will explore, using the light shed on the issue by previous conceptualizations, three concepts: gamification, game, and game experience. The characteristics that define each of them will be explained; later, we will define and characterize competitive debate, the new training model outlined here, in order to ascertain whether it may be considered to be gamification. To this end, we have performed a bibliographical analysis of the concepts of gamification, game, and game experience, and of their characteristics, as well as of the concept of debate itself.

## Concept Review

In this part, concepts that derive from the gamification concept and offer a deeper view (game and game experience) are reviewed. Later, various competitive debate definitions will be explained.

**Figure 1:**
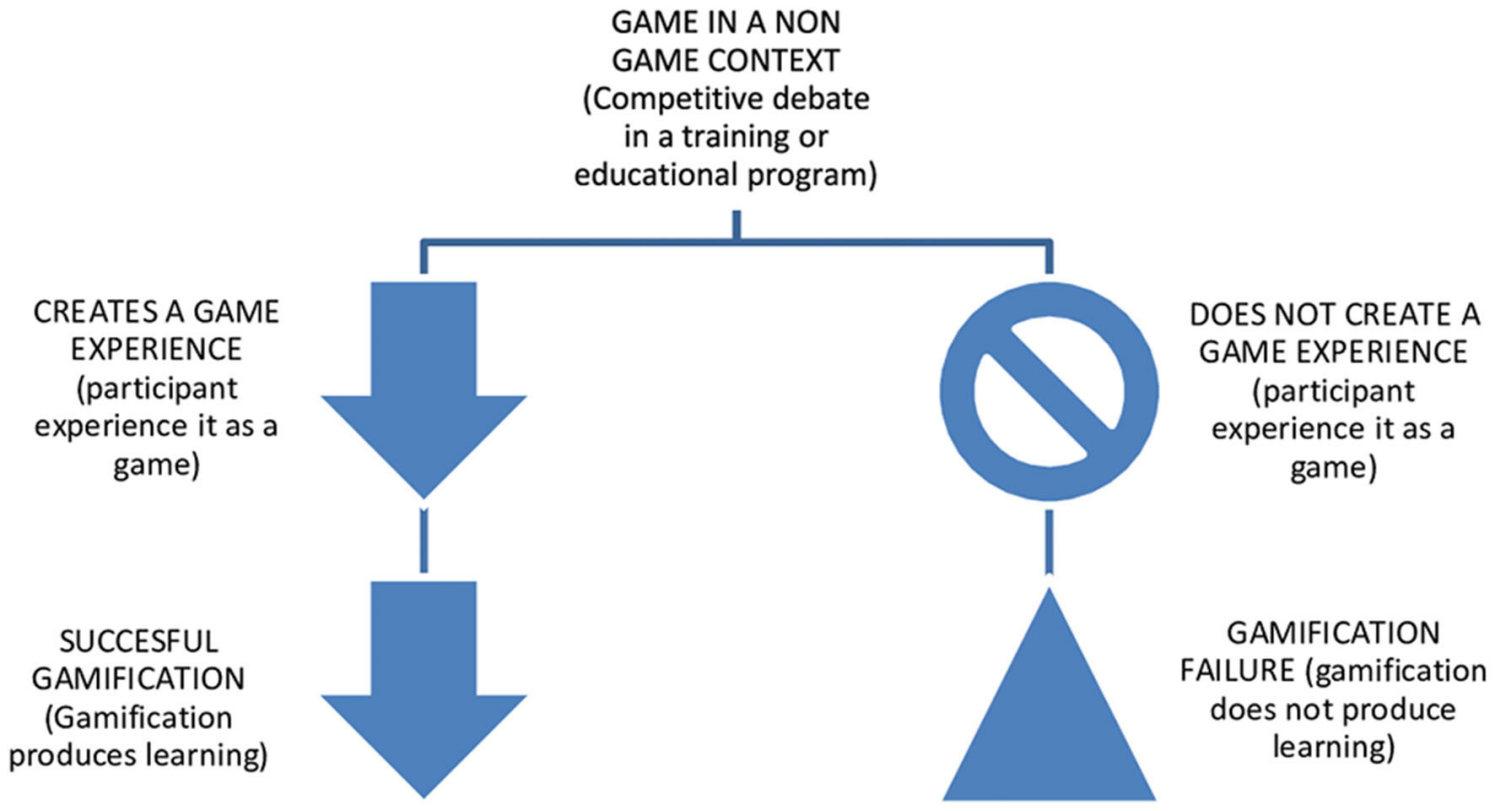
Relationship flowchart between game, game experience and gamfication for learning. *Source*: Own development.

The starting point for this work is the question of whether debate enters the field of gamification. The logos of the present work is that, in order for gamification to exist, there must be a game, and in order to consider such to be a game, there must exist a game experience. Therefore, if academic discussion decides in the affirmative, it may be concluded that debate is part of gamification. Figure 1 visually explains which of the concepts is subordinate to which.

In answering the question from a conceptual perspective, the team used Google Scholar to research publications that feature definitions of gamification, game, and game experience, and their characteristics. The motive for using this platform is that its files search is wider than others such as, for instance, the Web of Science or Scopus (Delgado and Repiso, 2013), since Google Scholar includes a great deal of gray literature.

In accordance with the logos and the findings, the corpus structure of this article is the following:

1. Current gamification definitions, game, and game experience are presented, and their essential characteristics are defined.
2. Differing competitive debate definitions are explained.
3. The characteristics of a gamification training tool, game experience, and game are analyzed so as to determine to what extent competitive academic debate is suited to them, and therefore whether debate is a gamifiable technique. The following criteria were considered when selecting gamification definitions: (1) literature written in English; (2) not exclusively computational gamification definitions; (3) education-related papers; and (4) definitions, preferably deriving from meta-analysis.

As for the selected definitions of game experience, game, and gamification, they were drawn, rather than from generic definitions, on the basis of which of their implementation features can be extracted, so as to check whether these features do in fact occur in the context of competitive debate. At the same time, we decided to select those characteristics of definitions that were not used in similar works (Bartanen and Littlefield, 2015), who take two authors to select their game definitions (Gray, 2008; Eberle, 2014) and then determine whether the debate meets the requirements that allow it to be considered a game. Our aim is that such an approach will allow for a more original analysis.

### Gamification and Related Concepts: Game Experience, Game, and Concept Review of Competitive Debate

In order to proceed with this conceptual analysis, it is important to clarify that the logos that structures this section is based on the following *matrioska* sequence: the concept of “gamification” contains or needs the concept of “game,” and the latter the concept of “game experience.” At the same time, our aim is to find out whether or not competitive debate fits the characteristics of the three concepts. The idea is illustrated in Figure 1.

### Game Experience

Rapp et al. (2019) question whether “the system is really producing an enjoyable and engaging experience…” when designing gamified systems. Because of that, and because the definitions of gamification do not fully answer the question of whether debate is gamifiable, it is necessary to expand our search and seek terms such as “game experience.” According to Deterding et al. (2011), the essence of a game is not only in a game's system, rules, and application, but also in the experience of the game itself. This experience is subjective, starting from the moment a person may or may not experience the sensation of play. Thus, for a game to be considered such, it must be considered a game by the person who plays it (Landers et al., 2019; p. 3). Landers defines the game experience as the psychological state resulting from the interaction of three psychological characteristics: perception of goals, goal-directed motivation, and a volitional attitude. Landers adds that if one of these characteristics is absent, it is not possible to talk about game experience. He thus comes, after analyzing the literature, to the conclusion that three characteristics must be present to confirm that players have had a game experience: (1) the players must perceive non-trivial but achievable goals that establish a certain degree of challenge and conflict that, in the end, lead to a measurable result; (2) the players will be motivated to pursue these goals under external arbitrary restrictions that lead to a quantifiable result defined by the rules of the game; and (3) the decision of the players to pursue these goals, under the restrictions, is taken voluntarily. Later, Landers clarifies that, if the player feels free to stop playing, the act of the players is, as a consequence, volitional.

Landers cites other cases in which the use of game elements does not render a game an experience, coinciding with Shpakova et al. (2019). The use, for example, of elements such as points, honors, or leaderboards, out of context, does not achieve the desired results in terms of gaming experience (Landers et al., 2019).

The characteristics of game experience definitions are presented in Table 1.

**Table 1:** Characteristics of the concept game experience.

**Characteristics of the game experience concept**	**References**
Player must exert some effort	Landers et al., 2019
Predetermined goals	
Artificial nature of the game	
Challenge and involve participants	

*Source: own elaboration*.

### Game

Before analyzing the concept of a game, it is necessary to differentiate play and game. The former, play, refers to the act of playing itself. The second one, game, targets a series of structured rules. This is the difference between *paidia* (free, expressive, and improvised) and *ludus* (structured, with rules, competitive, ordered to goals) (Deterding et al., 2011). Likewise, Landers et al. (2019) explains that playing (play) is the instinctive way in which children relate to the world. Children's play is characterized by its fluidity and the lack of specific goals.

Regarding the concept of game, Sauvé et al. (2007) argue that its characteristics are that there must be: player or players, a conflict, rules, a predetermined goal, and that the game be of an artificial nature. The definition of game by Gray (2008), drawn from Bartanen and Littlefield (2015), states that (a) the game be self-directed and (b) chosen by the person who plays it; (c) the means are more highly valued than the end; (d) there are rules that emanate from whoever plays; (e) it is imaginative; (f) it is not something truly serious; and (g) the game requires a mental state of alertness, but not stress. Juul (2010), taken from Deterding et al. (2011) states that “a game is a formal system based on rules with a variable and quantifiable result where different results represent different values, the player exerts effort in order to influence the result, the player feels tied to the effort, and the consequences of the activity are optional and negotiable.” From this definition it is clear that we are speaking of a feeling linked to effort, and that the psychological element is once again present. The definition of Eberle (2014), taken from Bartanen and Littlefield (2015), presents six characteristics: anticipation, surprise, pleasure, understanding, effort, and balance. Landers et al. (2019, p.7) finds several aspects common to all definitions of the game, namely that every game has a systemic approach to how the game itself is constructed, and that every game has an experiential component that requires the involvement of at least one player.

The characteristics extracted from the game definitions appear in Table 2.

**Table 2:** Characteristics of the game concept.

**Characteristics of the concept game**	**References**
Formal system based in rules	Sauvé et al., 2007; Juul, 2010
Result is quantifiable and objective	Juul, 2010
Result is variable	
The player has to do some effort	
It is suitable for social and cooperative use. There is competition and collaboration	Romero et al., 2012
*Storytelling* or narrative that structures the game	Girardelli et al., 2016
Players or player	Sauvé et al., 2007
Conflict	
Predetermined goal	
Artificial nature	
Challenge and involve the participant	De Freitas, 2006

*Source: own elaboration*.

### Gamification

Examples of gamification are present in multiple online areas. We have, for example, the autonomous language-learning application Duolingo (Huynh et al., 2016), in which, *via* games and points achievement, language students raise their level in line with given objectives. Also online, the website Kahoot features differing testing possibilities in which participants win points whilst competing with the aim of winning a knowledge-based competition. In the banking field, applications such as the BBVA Game helps clients to relate in a playful manner to digital banking. Beyond the online environment, there exists field training, in which different teams within an organization compete outdoors to achieve objectives and win a competition (Wagner et al., 1991).

For Hammer and Lee (2011) gamification “is the use of game mechanics, dynamics, and frameworks to promote desired behaviors”: the term “behaviors” might encompass, say, the act of purchase, such as the use of gamification aimed at increasing sales, or the successful mastery of professional communication skills. Unlike other definitions, this statement of the term focuses more on the purpose of the game.

For Deterding et al. (2011) gamification refers to “the use (rather than the extension of) of design (rather than game-based technology or other game-related practices) elements (rather than full-fledged games) characteristic for games (rather than play or playfulness) in non-game contexts (regardless of specific usage intentions, the contexts in which it is intended to be used, or the means of implementation).” Studies by Zainuddin et al. (2020) as well as de Sousa Borges et al. (2014) also point in this direction. What is certain is that the use of isolated elements of gamification does not convert the experience of gamification into an authentic game experience, as other authors concur (Landers et al., 2015; Shpakova et al., 2019).

Gamification is defined as “the use of the mechanics, aesthetics, and ways of thinking of the game to engage people, motivate them to action, and solve problems” (Kapp, 2012b). Kapp, initially, focuses the study on the use of gamification in video games, but later extends it to different areas. Other experts on gamification (Deterding et al., 2011) argue that “Although the overwhelming majority of current examples of “gamification” are digital, the term should not be limited to digital technology. Not only are media convergence and ubiquitous computing increasingly blurring the distinction between digital and non-digital: games and game design are themselves transmedial categories.” Another definition of gamification is “transforming activities, systems, services, products, or organizational structures to afford gameful experiences” which justifies and relates to the concept of game experience (Klock et al., 2020).

All the characteristics identified in analyzed gamification definitions are shown in Table 3. This table summarizes elements rather than definitions or concepts so as to establish whether or not those elements are applicable to debate.

**Table 3:** Characteristics of the gamification concept.

**Characteristics of the concept gamification**	**References**
Use of game mechanics or dynamics or elements	Deterding et al., 2011; Hammer and Lee, 2011; Kapp, 2012a,b
Clear start and end	
Freedom to make mistakes	Girardelli et al., 2016
Immediate feedback (result)	Jarvis and De Freitas, 2009; Pastor et al., 2015; Girardelli et al., 2016

*Source: own elaboration*.

In this section, we have extracted the characteristics of the three concepts that structure this approach: gamification, game, and game experience. Subsequently, in Table 4, the three concepts are placed in the context of competitive debate so as to check whether it complies with the characteristics of the above-mentioned concepts and whether, therefore, debate may fall under the umbrella definition of “gamification”:

**Table 4:** Characteristics of gamification/game/game experience related to competitive debate.

**Characteristic of the concept and origin: (gamification/game/game experience)**	**References**	**Present in competitive debate**	**Relation with competitive debate**
Use of game mechanics (Gamification)	Hammer and Lee, 2011; Kapp, 2012b	Yes	Someone wins or loses according to the jury or third party.
Formal rule-based system (Game)	Sauvé et al., 2007; Juul, 2010	Yes	Always starts and closes the affirmative, there is a third part who appoints the winner, there are fixed rules for times, etc.
Quantifiable and objective result (Gamification) (Game)	Juul, 2010; Landers et al., 2015	Yes	According to the debate format, the score may be by points or simply winner and loser. The result is always quantifiable.
There are clear start and a finish (Gamification)	Kapp, 2012b	Yes	It begins and ends by the same debater who starts with an initial presentation and closes the debate with a final refutation. The usual rule is that the affirmative side who supports the resolution starts. The duration time is usually fixed, which limits the beginning and the end.
Variable result (Game)	Juul, 2010	Yes	Anyone can win affirmative or negative
Players have to exert some effort (Game) (Game experience)	Juul, 2010; Landers et al., 2019	Yes	Players must present arguments, prepare them, present them and make them persuasive besides refuting the other side. We have to add the fact of public speaking and being evaluated by a jury.
Freedom to make mistakes (Gamification)	Girardelli et al., 2016	Yes	The argumentative and refutation strategy is left to the decision of the debater.
Immediate feedback (result) (Gamification)	Jarvis and De Freitas, 2009; Pastor et al., 2015; Girardelli et al., 2016	Yes	The result can be communicated at the end of each debate and is practically instantaneous according to the debate formats.
Suitable for social and cooperative use, there is competition and collaboration (Game)	Romero et al., 2012	Yes	Learning necessarily occurs when working in a team and also when a debater interacts with another debater. Whether it is online or face-to-face, individual or team debate, there is always social interaction.
Storytelling (Game)	Girardelli et al., 2016	No	It does not occur in the debate in backbone terms as would be the case of the videogames, although it is used as an element of persuasion in the debate.
Players or player (Game) (Game experience)	Sauvé et al., 2007	Yes	They take place in different debate teams and/or debate turns: initial presentation, rebuttal, counter-rebuttals and conclusions.
Conflict (Game)	Sauvé et al., 2007	Yes	There is a side for and another against a previously known resolution or there is a government and an opposition in the case of parliamentary formats.
Predetermined goal (Game) (Game experience)	Sauvé et al., 2007; Landers et al., 2019	Yes	The objective is to convince the jury or third party that my arguments are true and thus get their vote.
Artificial nature (game) (game experience)	Sauvé et al., 2007; Landers et al., 2019	Yes	An academic debate is an artificial situation, since it has to be organized.
Pedagogical nature (game)	Sauvé et al., 2007	Yes	The purpose of the educational field is to develop communication skills, persuasion, critical thinking, knowledge, etc.
Challenge and involve the participant (game) (game experience)	De Freitas, 2006; Landers et al., 2019	Yes	The answer to the debate resolution has to be justified. The participant faces the challenge of building his argumentation and attacking that of the opponent in order to convince the jury. The answer to the resolution must be justified.

*Source: own developement*.

Among these three concepts of the theoretical framework there are relationships of mutual influence, as well as differences. Between games and gamification, it is observed that while the aim of the game is recreational, in gamification the aim is not to have fun or enjoy oneself, but it is a means to another end: learning, changing habits, reorganizing systems, etc. The main difference is whether the game is an end or a means. As regards the similarities, in both concepts the game is present as a system of challenge to the participant with rewards, rules, and a legal system.

Regarding the relationship of game and game experience, the game is itself objective in terms of the rules and the result. On the contrary, the game experience is totally subjective as it depends on the psychological state of the participant. Each of these concepts requires the other, since if there is no psychological state of gaming experience, there can be no real game for the participant and, as we will see, it would not be appropriate to talk about gamification without also talking about game experience.

Regarding game experience and gamification, the former is a psychological state dependent on who experiences it or not, while the latter is a set of rules and rewards aimed at influencing people and organizations. In terms of their similarities, gamification needs to provoke that psychological state of gaming experience to influence people, otherwise, participants would abandon the game and therefore gamification would have no opportunity to influence the person or organization. These relationships are summarized in Table 5.

**Table 5:** Differences and confluences between game, game experience and gamification.

	**Differences**	**Confluences**
Game- gamification	- In a game, the objective or purpose is recreational, while in gamification the aim is to modify behaviors or systems. - The game is an end in itself in playful environments, the game is a means in gamified environments.	- In both cases the game is present with its rules and rewards systems.
Game- game experience	- The game is governed by a set of objective rules, unlike the game experience, which is the perception of persons, and therefore has a subjective component.	- There is a causal relationship between the game, a set of objective rules, which can result in a subjective psychological state.
Game experience- gamification	- The gaming experience is a psychological state, gamification is a system for modifying and influencing people and systems	- For gamification to respond to the objectives for which it is used successfully, it must coexist with the gaming experience

*Source: own developement*.

Among the three concepts, the relationship is that they need each other in the following sequence: a game is presented in a non-game context, and it creates, or not, a game experience and, depending on that result, gamification might be successful or not. This cycle is explained in Figure 1.

### Gamification Applications

Gamification is applied with differing purposes and in differing areas, including the educational, therapeutic, and business fields. In therapeutic settings, gamification applications have been designed for mobile and web with uses aimed at socio-emotional, physical, and cognitive well-being (McCallum Simon, 2012). In particular, the use of gamification has been applied to the treatment of depression (Dias et al., 2018) as well as to the treatment of addiction to video gaming, social networks, and digital elements in general (Jiang et al., 2015). Gamification is becoming a trend in the field of business management across different departments and functions, as well as in various sectors of business activity (Wanick and Bui, 2019), with gamification applications ranging from personal productivity to customer loyalty. Elements such as points allocation to frequent travelers in airlines or gas stations are also considered gamification (Sengupta and Sengupta, 2015).

Thus gamification is applicable to marketing, customer loyalty, user experience, personal productivity, and training. Likewise, gamification is used in recruitment, selection, training, and in various people management processes (Landers et al., 2015). But, according to Kapp (2012b), gamification is used more frequently for things such as security policies, product details, customer service, welcoming new hires, and information that employees need to remember periodically. According to the author, and in the light of the connotations given in the definitions, the definitions themselves present a wide variability that is conditioned by the diversity of uses and applications. It can thus easily be verified that there is neither one single definition of gamification nor one exclusive application of it.

### Competitive Debate Concept

The precise origins of debate are difficult to date. There are references to its pedagogical applications in ancient Greece with Protagoras of Abdera (Cirlin, 1999); Saint Thomas Aquinas created the *quaestiones disputatae de veriate*, in which his disciples debated controversial issues and practiced their skills of persuasion (Ortega de la Fuente, 2005). Likewise, in eighteenth-century Spanish universities, debates were a local spectacle followed the by non-academic population (Mercadal, 1934). In the United States, at the beginning of the twentieth century, academic debates were a public event until the emergence, in the 30 s, of sports competitions, which diminished the prominence of University debates (Bartanen and Littlefield, 2015).

Regarding debate applications, in addition to the extra-academic competitive school and University debate tournaments, academic application is given as a teaching and learning method (Mitchell, 1998; Roy and Macchiette, 2005; Yasuko, 2003; Takanokura and Hayashi, 2008; Algarra, 2015; Merrell et al., 2017; Sapitri, 2017; Galiano-Coronil et al., 2018). In the professional field and as far as is covered by the bibliographical survey, we have evidence only of the experience of Benton (2012). This would be a clear example of gamification according to our criteria here. A game, competitive debate, is taken into a non-game context, for example a University or school classroom.

The positive effects of the practice of debate on intellectual research capacities have been analyzed (Pernecky, 1997) along with its effects on fostering critical thinking (Colbert, 1987; Allen et al., 1997; Bellon, 2000; Roy and Macchiette, 2005; Perandones et al., 2018) and even as a mechanism of social mobility (Bartanen and Littlefield, 2015).

The debate and personal communication professor at Saint Mary's University, Alan Cirlin, offers no explicit definition of debate, but he does distinguish it from the concept of argumentation with persuasive purposes: “we argue with someone to convince him [sic], and we debate against an opponent to convince the audience.” (Cirlin, 1999). On the contrary Cattani (2003) defines debate as: “A competition (a challenge) between two antagonists, in which, unlike what happens in a simple discussion, there is a third party (a judge, an audience) whose approval is sought by the two contestants. It may be debated, even on issues that are considered impossible to resolve with the objective of persuading others.” The third party, judge or audience, and its approval, is emphasized. In an everyday argument, we try to convince our interlocutor, but this is not the case in an academic debate, in which a judge or jury must be convinced, or in an electoral debate, in which it is the undecided voter who must be persuaded. For their part, Huber and Snider (2006) explain that a debate “is the process of presenting arguments in favor of or against a proposition” They do not add more, although they make it clear that there must be a part in favor (or affirmative side) and another, against (or negative side) the debate resolution and that there is a clear process for the presentation of the arguments.

To establish a basic competitive debate, according to Cirlin (1999) a series of essential elements is necessary, namely a proposal or resolution, an affirmative side, which defends that the resolution is true, and a negative side, which denies that the resolution is true. There must also be a format of turns and times.

### Characteristics of Gamification, Game, Game Experience, and Its Relation to Competitive Debate

We will now present, in line with current research, the characteristics of gamification, game, and game experience. Our aim is to evaluate whether competitive debate fulfills these characteristics, and if so how.

In Table 5 the information has been arranged as follows. Column 1 displays the characteristics of each concept (gamification, game, or game experience). Column 2 describes which author or authors this characteristic is sourced from. In column 3 we note whether or not each characteristic is fulfilled by competitive debate. And finally, Column 4 explains why debate fulfills or not each requirement of Column 3, and to what extent.

Next, we discuss the relationship of each characteristic in respect to the concepts of debate gamification, game, and game experience.

#### Use of Game Mechanics

In every debate there is a winner and a loser. This consideration depends on the jury previously appointed for such a function (Cattani, 2003). According to Kapp (2012b), winning or losing is one of the characteristics of the competitive game, just as it is in a competitive debate.

#### Formal Rule-Based System

A competitive debate is always subject to rules, which, depending on the competition system applied, may be more or less rigorous and exhaustive. Cirlin (1999) presents different types of debate format in which elements such as time, intervention orders, penalties, scores, and judge decision methods are all established. Cirlin himself notes that there may be as many debate formats as may be deemed appropriate. The truth is that any debate is always based on rules; in a competitive debate, you never simply start debating. The use of formal rules systems is a key element of games (Sauvé et al., 2007; Juul, 2010).

#### Quantifiable Result

This is an overlapping characteristic of games and gamification (Juul, 2010; Landers et al., 2015). Although Cirlin (1999) establishes that the debate—or more the assessment or judgment of it—is a subjective activity, the result is objective. Sometimes the result simply declares a winner or a loser, though sometimes a more refined score may be given, depending on how the jury reaches its decision.

#### There Is a Clear Start and Finish

To Kapp (2012b), this is an essential element in both gamification and games. In a competitive debate, depending on the format, the duration may be longer or shorter. However, every debate begins and ends within a pre-established time frame, with experts in debate suggesting that formats range from 30-90 minutes maximum (Cirlin, 1999).

#### Variable Result

Juul (2010) states that a variable result is a basic characteristic of games. The debate outcome, in terms of winner or loser, is unpredictable. Only the jury may reach a verdict, since it is the third part that the debaters accept as decisive when the winner is declared.

#### The Players Have to Exert Effort

This is a common element in both game and game experience (Juul, 2010; Landers et al., 2019). The player (in this case the debater) has to develop an argument, persuade judges through their communication skills, verbal and non-verbal, and use quotes, evidence, active listening, and other strategies in order to attack the arguments of the opponent and to capture the attention of jury and audience. Ultimately, the effort is both intellectual and, when it comes to dealing with the stress of public speaking, emotional.

#### Freedom to Make Mistakes

This characteristic comes from Girardelli et al.'s (2016) work. The debater decides which arguments to use, which to attack, and whether to make use of one particular linguistic register or another. The participant must thus constantly take decisions, so there is also the freedom to make mistakes.

#### Immediate Feedback

This is an essential element of gamification (Jarvis and De Freitas, 2009; Girardelli et al., 2016; Pastor 2018). The result of who is designated winner is what some authors mean by feedback. In a debate tournament, the jury communicates the result, either to the participants or to the tournament organizers, after each debate, so that the result is immediate. “Feedback” might be taken to mean either the result of winning or losing, or the critical appraisal of the debate performance, but when it comes to competitive debate both can occur immediately.

#### Appropriately for Social and Cooperative Use, Both Competition, and Collaboration Are Present

This characteristic from Romero et al. (2012) is in line with competitive debate. In a debate, especially if it is in a team, one must necessarily cooperate in order to debate. Sánchez Prieto (2017) shows that a classroom debate is suited to all active learning methodologies and, more specifically, to cooperative learning. On the other hand, it is impossible to debate with oneself, meaning that social interaction is key.

#### Storytelling

According to Girardelli et al. (2016) storytelling is a characteristic of games. Debate does not fulfill the criteria of having a narrative that structures the game, since, unlike a video game or roleplay game, there is no narrative to act as a through-line. Despite this, storytelling might be present as a persuasive technique.

#### Players

Regardless of whether it involves teams or individuals, the role of player must be performed (Sauvé et al., 2007). The debaters themselves fulfill the role of competitors or players.

#### Conflict

A clear conflict must exist as a starting point for a game (Sauvé et al., 2007). In competitive debate one part must convince the jury that the resolution is true and, the other, that the resolution is false: this describes the conflict that arises in a debate. In fact, according to Huber and Snider (2006), the debate system is based on the United States court hearing system, a tried and tested way of resolving conflicts.

#### Predetermined Goals

To be considered a game there must be predetermined goals (Sauvé et al., 2007). In a competitive debate, the mission of each side is determined when it is assigned as defending the affirmative or negative side: the affirmative side has the goal of persuading the jury that the resolution is true and, in the case of the negative position, that the resolution is false. Both parts must convince the jury that it is they who is best defending their position. Regarding games, Landers et al. (2019) add that rules have to be more limiting than they normally would be. Thus, in a debate the times are fixed and limited, and one cannot speak or interrupt as one pleases, unlike what might occur in a normal discussion or conversation.

#### Artificial Nature

A common element to game and game experience (Sauvé et al., 2007; Landers et al., 2019) is the artificial nature of the situation. Debating in order to compete is not a natural situation, since the debate resolution to a specific issue is programmed and agreed upon and even restricted to a specific question. Moreover the psychological state that instigates a game or game experience is unique.

#### Pedagogical Nature

This characteristic of games is particular to games, but also to certain gamification approaches (Sauvé et al., 2007). For our model, since the ultimate objective is to bring debate into professional organizations in order to develop communication, creative thinking, and other skills, it has a clear pedagogical and training orientation.

#### Challenge and Involvement of the Participant

This element is common to game and game experience (De Freitas, 2006; Landers et al., 2019). Participation in a debate involves the debater through the obligation of answering the question for which they are responsible. The challenge also falls on different axes: answering the question, affirmatively or negatively, in a way with which the debater possibly does not agree; counteracting the lines of the opposing team; and finally convincing the jury of all of the above, and that the debater's argumentation represents the correct view and that the opponents' does not. All these functions represent challenges for the debater.

## Conclusions

The article enquires into whether competitive debate is, or is not, a game exercise and whether it is likely to be considered a gamification mechanism. To investigate this, an analysis of the concepts of gamification, game, game experience, and debate is used, extracting the characteristics of each element and then checking whether competitive debate for adult learners harbors such characteristics. We can affirm, according to our consulted authors, that there is no unanimity regarding the definitions of either gamification, nor game, or in the elements that compose them. There do exist common elements between gamification and game, and between game and game experience, but not, in the light of the consulted literature, among all three.

Regardless of the authors and the reviewed approaches—gamification, game, and game experience—we can conclude that debate can indeed be framed in the category of gamifiable technique, as it meets all the criteria of gamification, game experience, and game. Of the elements analyzed, all are met except that of storytelling, or the implicit narrative that may govern the course of the game (in a story or narrative such as those found in videogaming). This characteristic does not occur in competitive debate, because there is no narrative *per se* that rules the order of the game, although sometimes stories are used to persuade a jury. Competitive debate may therefore indeed be considered a game, as Bartanen and Littlefield (2015) assert, as well as a gamification exercise, as long as it is applied for educational or training purposes, with a game experience being created around the debate.

If, as certain authors have concluded, (Deterding et al., 2011; de Sousa Borges et al., 2014; Klock et al., 2020), gamification consists of applying games in non-game environments, when applying competitive debate, which is a game (Bartanen and Littlefield, 2015) to these non-game environments (such as a classroom in educational institutions or training programs in a company), it can be inferred that, as we have attempted to show here, competitive debate in non-game contexts may also be included under the category of gamification.

## Limitations and Future Lines of Research

Research has not yet clarified whether or not debate is actually taken to be a game by the participants themselves; that is to say, whether the participant's psychological state is similar to that in games, based on what Landers et al. (2019) affirm. The perception of the experience and the sensation of learning should also be investigated. Likewise, the issue of return on investment remains open in terms of debate as a training tool, insofar as it is more effective and efficient when training professionals. An experimental design platform could be configured, and an analysis of the debate model implementation in business training could be performed, with the specific aim of teaching communication abilities and problem-solving skills. In this research, two dimensions would be measured—on the one hand the evaluation, *via* a questionnaire, of the participant's own experience, and on the other an evaluation of their learning in communication skills, by using external evaluators, who would observe the scope of the learning according to a previously-validated tool, much as Benton (2012) requires in his work on debate, diversity, and adult education.

One limitation is that a specific term for non-digital gamification is not found in the literature. Most of the research literature does not specifically name non-digital gamification, despite the demands of the scientific community to research into this type of gamification. A specific term for non-digital gamification would render search and analysis for this type of training solution more operational.

In general, an inductive-deductive scheme is needed to reach more robust conclusions as to whether competitive debate fits into the different concepts of game and game experience, for which a series of empirical and experimental developments would be necessary.

As a research gap, it remains to be discovered in which skills (public speaking, persuasion, decision-making, problem solving, etc.) and fields of knowledge (procedures, areas of knowledge, etc.) the use of competitive debate is most relevant. In this sense, there is room for research in both the University and the professional field. Meanwhile, the application of debates as an organizational solution for meetings, personnel selection, or talent detection could also be the focus of research. It is to be hoped that this paper may open a new line of research that could be of assistance to both professional organizations seeking to discover new training dynamics and to gamification itself, with the subsequent creation of new applications to test, and effects to discover, on behaviors, on engagement, and on knowledge acquisition.

## Author Contributions

All authors conceived the research, designed the article, and developed all the tables and figures used in this article.

## Conflict of Interest

The authors declare that the research was conducted in the absence of any commercial or financial relationships that could be construed as a potential conflict of interest.

## Publisher's Note

All claims expressed in this article are solely those of the authors and do not necessarily represent those of their affiliated organizations, or those of the publisher, the editors and the reviewers. Any product that may be evaluated in this article, or claim that may be made by its manufacturer, is not guaranteed or endorsed by the publisher.
